# Satellite Cells Derived from Obese Humans with Type 2 Diabetes and Differentiated into Myocytes In Vitro Exhibit Abnormal Response to IL-6

**DOI:** 10.1371/journal.pone.0039657

**Published:** 2012-06-26

**Authors:** Camilla Scheele, Søren Nielsen, Meghan Kelly, Christa Broholm, Anders Rinnov Nielsen, Sarah Taudorf, Maria Pedersen, Christian P. Fischer, Bente Klarlund Pedersen

**Affiliations:** The Centre of Inflammation and Metabolism at Department of Infectious Diseases and Copenhagen Muscle Research Centre, Rigshospitalet, The Faculty of Health Sciences, University of Copenhagen, Copenhagen, Denmark; Université Joseph Fourier, France

## Abstract

Obesity and type 2 diabetes are associated with chronically elevated systemic levels of IL-6, a pro-inflammatory cytokine with a role in skeletal muscle metabolism that signals through the IL-6 receptor (IL-6Rα). We hypothesized that skeletal muscle in obesity-associated type 2 diabetes develops a resistance to IL-6. By utilizing western blot analysis, we demonstrate that IL-6Rα protein was down regulated in skeletal muscle biopsies from obese persons with and without type 2 diabetes. To further investigate the status of IL-6 signaling in skeletal muscle in obesity-associated type 2 diabetes, we isolated satellite cells from skeletal muscle of people that were healthy (He), obese (Ob) or were obese and had type 2 diabetes (DM), and differentiated them *in vitro* into myocytes. Down-regulation of IL-6Rα was conserved in Ob myocytes. In addition, acute IL-6 administration for 30, 60 and 120 minutes, resulted in a down-regulation of IL-6Rα protein in Ob myocytes compared to both He myocytes (P<0.05) and DM myocytes (P<0.05). Interestingly, there was a strong time-dependent regulation of IL-6Rα protein in response to IL-6 (P<0.001) in He myocytes, not present in the other groups. Assessing downstream signaling, DM, but not Ob myocytes demonstrated a trend towards an increased protein phosphorylation of STAT3 in DM myocytes (P = 0.067) accompanied by a reduced SOCS3 protein induction (P<0.05), in response to IL-6 administration. Despite this loss of negative control, IL-6 failed to increase AMPKα2 activity and IL-6 mRNA expression in DM myocytes. There was no difference in fusion capacity of myocytes between cell groups. Our data suggest that negative control of IL-6 signaling is increased in myocytes in obesity, whereas a dysfunctional IL-6 signaling is established further downstream of IL-6Rα in DM myocytes, possibly representing a novel mechanism by which skeletal muscle function is compromised in type 2 diabetes.

## Introduction

Type 2 diabetes is a condition of metabolic failure preceded by skeletal muscle insulin resistance. It is possible that concurrent resistance to additional growth factors or cytokines is established and contributes to the muscular phenotype of type 2 diabetes. One candidate protein involved in skeletal muscle metabolism, and also regulated during obesity and diabetes is interleukin (IL)-6. The role of IL-6 in skeletal muscle metabolism has been debated. IL-6 is markedly produced by contracting skeletal muscle and released into plasma during the post-exercise period [1], [2] when insulin sensitivity is enhanced [3]. In sharp contrast, adipose production and secretion of IL-6 [4] is chronically elevated in obese people [5], [6] and elevated plasma IL-6 is an independent risk factor for developing type 2 diabetes [6], [7]. Thus, it has been suggested that IL-6 contributes to obesity-associated insulin resistance [8], supported by the findings that increased plasma levels of IL-6 are associated with both obesity and insulin resistance [7], [9], [10]. However, it was recently shown that IL-6 stimulates insulin production by inducing Glucagon-like peptide-1 (GLP-1) expression [11]. This important finding suggests that the increased IL-6 production associated with obesity and insulin resistance may in fact represent a mechanism for increasing the production of insulin, an idea that warrant further investigation.

At the cell membrane, IL-6 binds to an IL-6Rα-gp130 receptor complex, activating JAK/STAT3 signaling. IL-6 also induces Suppressor of cytokine signaling 3 (SOCS-3) gene expression via JAK/STAT3 signaling, acting in a negative feedback loop. SOCS-3 interacts with JAK tyrosine kinase and inhibits phosphorylation of JAK substrates, such as the STAT proteins involved in cytokine signaling [12]. In addition, SOCS-3 has been described as an inhibitor of the phosphorylation of IRS-1 [13]. In line with this, increased SOCS-3 impairs glycogen synthesis and glucose transport [14], [15]. Thus, a dysregulation of the IL-6 signaling pathway involving a dysregulation of SOCS-3 could possibly be related to a deficient insulin signaling.

Skeletal muscle expresses IL-6Rα, which also increases with physical activity [16] suggesting an autocrine IL-6 signaling loop (2). In support of this idea, it was shown that IL-6 mRNA expression was induced in human skeletal muscle in response to IL-6 infusion [17], while *in vitro* administration of recombinant IL-6 induced IL-6 mRNA expression in the murine muscle cell line, C2C12 [18]. IL-6 has been shown to stimulate metabolic rate in skeletal muscle cells by increasing AMPKα2 activity [19], [20], fatty acid oxidation and glucose uptake [21]–[23].In order to model human muscle signaling *in vitro*, several studies have previously demonstrated that the so called satellite cells, which are muscle stem cells stored at the sarcolemma, can be isolated from muscle biopsies and differentiated into myotubes *in vitro*. Interestingly, it has also been shown that muscle precursor cells isolated from skeletal muscle biopsies from individuals with type 2 diabetes, have established donor-specific phenotypes, which transpires following differentiation into myocytes *in vitro*. So far, it has been demonstrated that myocytes derived from people with type 2 diabetes have reduced insulin sensitivity and reduced glucose transport [24]–[27], reduced lipid oxidation [28] and an increased activity of inflammatory markers [27].

Interestingly, IL-6 has been shown essential for muscle hypertrophy to occur through activation of STAT3 signaling in murine satellite cells [29]. In contrast, other studies report a muscle atrophic effect of long-term exposure of IL-6 [30], [31]. Thus, chronic exposure of elevated levels of IL-6 might result in a dysregulation of IL-6 signaling in skeletal muscle cells.

Our aim with this study was to investigate whether IL-6 signaling is affected in skeletal muscle or in satellite cells of people with type 2 diabetes. We hypothesized that satellite cells derived from people with type 2 diabetes would demonstrate a deficient response to IL-6, which could contribute to the metabolic dysfunction of skeletal muscle in type 2 diabetes. We measured the abundance of the IL-6 receptor (IL-6Rα) in skeletal muscle biopsies and isolated satellite cells from healthy (He), obese (Ob) or obese persons with type 2 diabetes (DM). These cells were differentiated *in vitro* and utilized as a model to study IL-6 signaling in human skeletal muscle. We achieved our aim and identified a down-regulation of IL-6Rα in obesity and an abnormal IL-6 signaling in myocytes from people with type 2 diabetes.

## Results

### Dysregulation of IL-6Rα in Myocytes of People with Obesity

**Figure 1:**
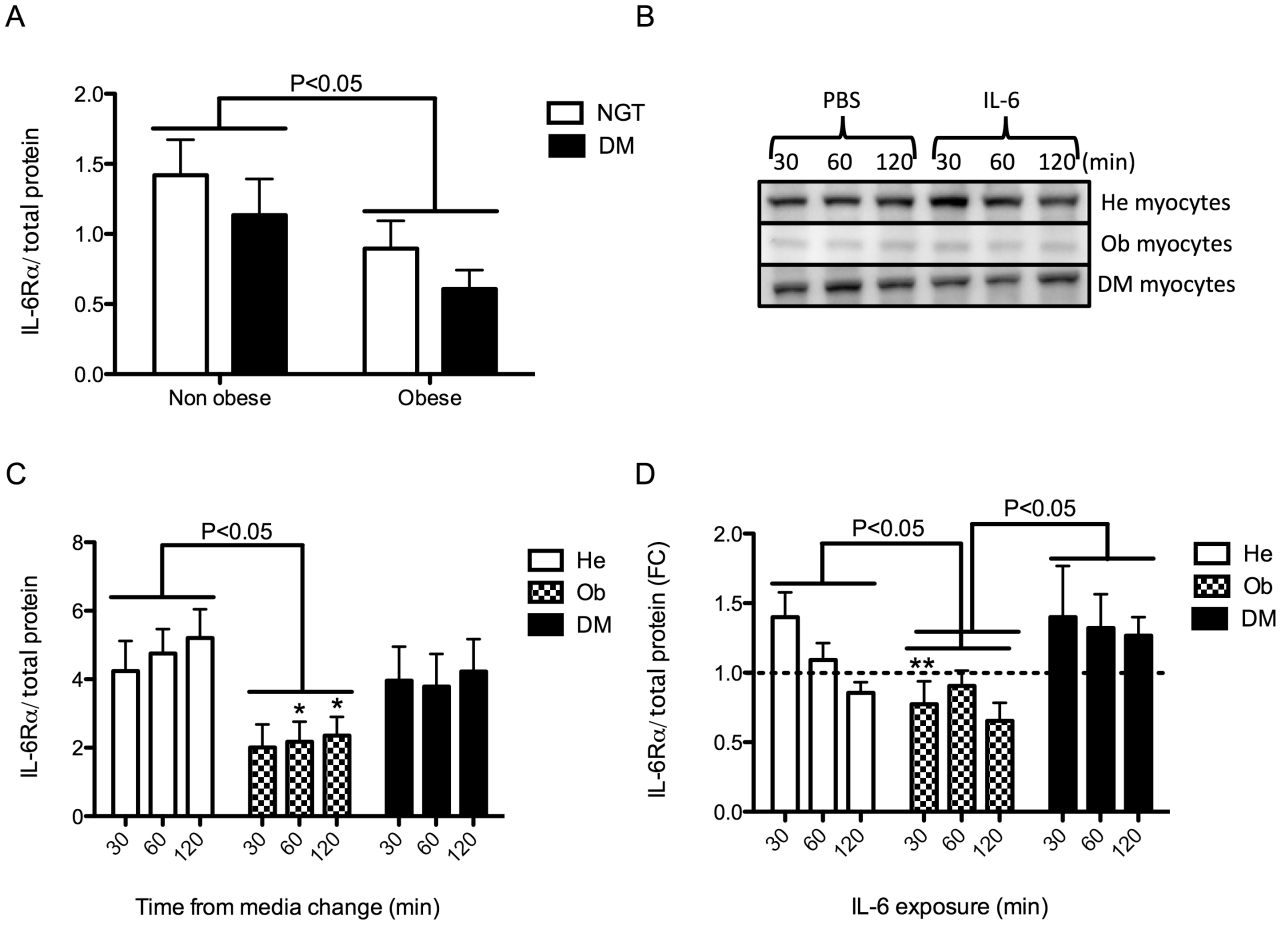
IL-6Rα protein expression in skeletal muscle tissue biopsies and in He, Ob and DM myocytes in response to IL-6. (A) Protein expression of IL-6Rα was assessed in skeletal muscle biopsies from the vastus lateralis muscle of non-obese (n = 10) or obese (n = 10) normal glucose tolerant (NGT) subjects and non-obese (n = 10) or obese (n = 9) subjects with type 2 diabetes (Table 1), using western blot analysis. Due to the variability in these *in vivo* samples, all blots are presented in Figure S1. A two-way ANOVA was performed, comparing the effect of obesity and the effect of diabetes on IL-6Rα protein expression. (B) Protein expression of IL-6Rα was assessed in satellite cells isolated from healthy (He) (n = 7), obese (Ob) (n = 7) and obese people (n = 7) with type 2 diabetes (DM) (Table 2). Satellite cells were differentiated into myocytes and treated with IL-6 (100 ng/ml) or control (PBS) for 30, 60 and 120 min. (C) Baseline IL-6Rα protein expression was normalized to total protein expression (as determined by reactive brown staining) and compared between groups by two-way ANOVAs. (D) IL-6 induced IL-6Rα protein expression was normalized to total protein and compared between groups by two-way ANOVAs and effect of time within groups was assessed with one-way ANOVAs. Data are presented as fold change (FC) between the control samples presented in B and C and samples treated with IL-6 (100 ng/ml). Data are mean ± SE. Groups were compared in pairs (i.e. He vs Ob, He vs DM and Ob vs DM) by two-way ANOVAs. Results from ANOVAs are marked in the figures with connecting capped arcs. *Results from Bonferroni post-tests, relative to He myocytes; *P<0.05, **P<0.01, ***P<0.001.

To investigate whether a deficient IL-6 signaling was established in skeletal muscle of subjects with obesity or type 2 diabetes, we measured the protein abundance of IL-6Rα in skeletal muscle biopsies from normal glucose tolerant (NGT) subjects and people with type 2 diabetes that were either non-obese or obese (DM) (Table 1). Western blot and a two-way Anova demonstrated an effect of obesity (P<0.05) but no independent effect of diabetes (Figure 1A and Figure S1). We then investigated the IL-6Rα protein abundance in an *in vitro* model consisting of satellite cells isolated from healthy (He), obese (Ob) and people with type 2 diabetes (DM) (Table 2) and differentiated *in vitro* into myocytes. Interestingly, the down-regulation of IL-6Rα protein observed in tissue was conserved in Ob myocytes (despite being isolated from a different cohort) (P<0.05), but not in DM myocytes (Figure 1B and C). Furthermore, when stimulating the myocytes with recombinant IL-6 for 30, 60 and 120 minutes, IL-6 was strongly regulated over time in He myocytes with a trend towards an initial up-regulation at 30 minutes of IL-6 incubation, followed by a gradual, highly time-dependent down-regulation (P<0.001) (Figure 1D). In contrast, in Ob myocytes, the IL-6Rα protein expression in response to IL-6 was down-regulated already at 30 min of IL-6 incubation (P<0.01), compared to He myocytes, and were not further down-regulated over time. Finally, DM myocytes were not down-regulated at all but had an up-regulated IL-6Rα response to IL-6 when compared as a group to Ob myocytes (P<0.05). This data is intriguing as the diabetic cell donor subjects were also obese and in muscle tissue IL-6Rα protein was down-regulated in both obese normal glucose tolerant individuals and in obese individuals with type 2 diabetes. To investigate the potential consequence of the altered response observed in Ob and DM myocytes, we assessed downstream signaling.

**Table 1:** Subject characteristics for muscle biopsy protein analysis, human study 2.

Subjects	NGT non-obese	NGT obese	DM non-obese	DM obese
N	10	10	10	9
Gender	5 Male, 5 Female	5 Male, 5 Female	5 Male, 5 Female	5 Male, 4 Female
Age	56.2 (±9.5)	52.3 (±9.9)	61.7 (±12.9)	56.11 (±4.0)
BMI	25.3 (±2.1)	39.5 (±5.6)***	25.6 (±2.5)	37.5 (±5.9)***
0 h Glucose (mmol/l)	5.3 (±0.5)	5.2 (±0.3)	9.9 (±3.8) * $	12.2 (±5.7)*** $$$
2 h Glucose (mmol/l)	6.0 (±1.6)	6.4 (±0.8)	19.4 (±7.8) *** $$$	19.4 (±6.4)*** $$$

Bonferroni post-tests provided P values for normal glucose tolerant people with obesity (NGT obese) or type 2 diabetes (DM non-obese and DM obese) relative to normal glucose tolerant control subjects (NGT non-obese);

* P<0.05;

** P<0.01;

*** P<0.001. Values are mean ± SD. Bonferroni post-tests was also performed for DM obese relative to NGT obese and provided P values;

$ P<0.05;

$$ P<0.01;

$$$ P<0.001. Values are mean ± SD.

**Table 2:** Subject characteristics for primary muscle cell cultures, human study 1.

Subjects	Healthy	Obese	Type 2 diabetes
N	7	7	7
Gender	4 Male, 3 Female	4 Male, 3 Female	5 Male, 2 Female
Age	56 (±7.2)	51 (±5.7)	57 (±8.6)
BMI	26.1 (±2.0)	35.1 (±5.8)**	33.4 (±3.1)**
0 h Glucose(mmol/l)	5.7 (±0.6)	6.1 (±0.6)	8.7 (±2.4)** $$
2 h Glucose(mmol/l)	6.3 (±1.4)	8.2 (±0.7)	15.2 (±4.3)*** $$$

Bonferroni post-tests provided P values for people with obesity or type 2 diabetes relative to healthy control subjects;

* P<0.05;

** P<0.01;

*** P<0.001. Values are mean ± SD. Bonferroni post-tests provided P values for people with type 2 diabetes relative to obese subjects;

$ P<0.05;

$$ P<0.01

$$$ P<0.001. Values are mean ± SD.

### Dysregulation of IL-6 Induced STAT3/SOCS3 in Myocytes of People with type 2 Diabetes

**Figure 2:**
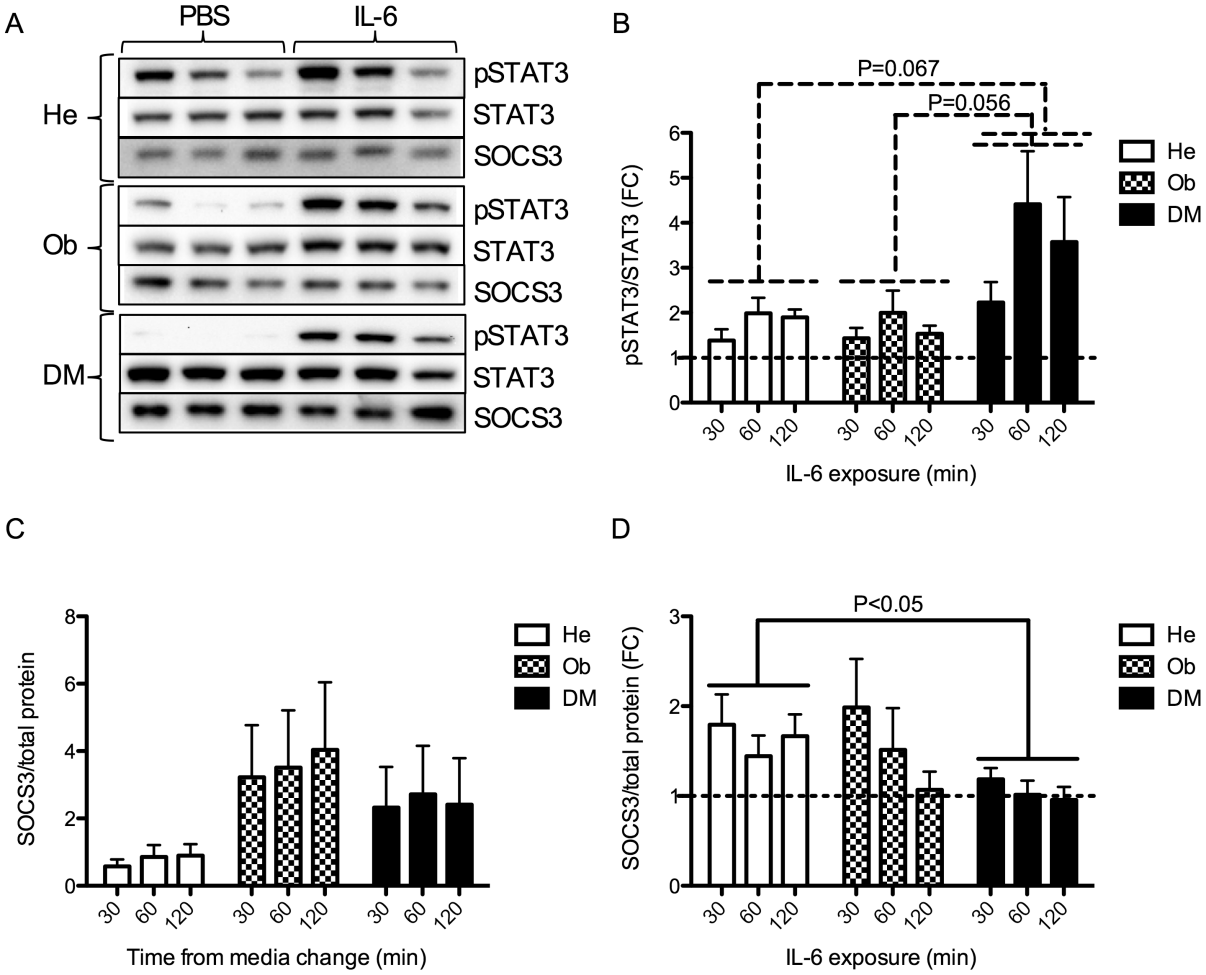
pSTAT3/STAT3 and SOCS3 protein expression in He, Ob and DM myocytes in response to IL-6. (A) Protein expression of STAT3, pSTAT3 and SOCS3 was assessed in satellite cells isolated from healthy (He) (n = 7), obese (Ob) (n = 7) and obese people with type 2 diabetes (DM) (n = 7) (Table 2). Satellite cells were differentiated into myocytes and treated with IL-6 (100 ng/ml) or control (PBS) for 30, 60 and 120 min (same protein samples as in Figure 1 B-D). (B) IL-6 induced phosphorylated STAT3 was related to total STAT3 levels and IL-6 treated samples were presented as fold change from PBS treated samples. IL-6 induction of pSTAT3 was compared between groups by two-way ANOVAs. (C) Baseline SOCS3 protein expression in the samples described in A was normalized to total protein expression (as determined by reactive brown staining) and compared between groups by two-way ANOVAs. (D) IL-6 induced SOCS3 protein expression was normalized to total protein expression and compared between groups by two-way ANOVAs. Data are presented as fold change (FC) between the control samples presented in A and C and samples treated with IL-6 (100 ng/ml). Data are mean ± SE. Groups were compared in pairs (i.e. He vs Ob, He vs DM and Ob vs DM) by two-way ANOVAs. Results from ANOVAs are marked in the figures with connecting capped arcs. *Results from Bonferroni post-tests, relative to He myocytes; *P<0.05, **P<0.01, ***P<0.001.

To investigate whether downstream IL-6 signaling was affected in the myocytes, we stimulated He, Ob and DM myocytes with recombinant IL-6 or PBS for 30, 60 and 120 minutes and measured downstream activation of STAT3 protein signaling, presented as phosphorylated STAT3/total STAT3 (Figure 2A and 2B). Interestingly, while we observed no difference between He and Ob myocytes, there was a clear trend towards an *increased* activation of STAT3 in DM myocytes compared to He myocytes (P = 0.067) and compared to Ob myocytes (P = 0.056) (Figure 2B). A possible mechanism for this observation could be a loss of control by the suppressor of cytokine signaling 3 (SOCS3). Thus, we measured protein levels of SOCS3 in the same samples as for pSTAT3/STAT3. Viewing the baseline control samples (stimulated with PBS only), there was a trend towards an increased SOCS3 expression in both Ob and DM myocytes, however without reaching significance (P = 0.138 for Ob myocytes and P = 0.238 for DM myocytes) (Figure 2C). However, when comparing the fold change of PBS and IL-6 treated samples between the groups, we observed a reduced SOCS3 protein up-regulation in response to IL-6 in DM myocytes compared to He myocytes (P<0.05) (Figure 2D). Interestingly, in response to IL-6, Ob myocytes seemed to activate STAT3 and SOCS3 to a similar level as did He myocytes, indicating that the dysregulation of IL-6 signaling at this level only occurred in DM myocytes (Figure 2B and 2D). Hence, these data indicate that DM myocytes, but not Ob myocytes, have increased pSTAT3 response accompanied by a reduced SOCS3 feedback control mechanism.

### Resistance to IL-6 Induced IL-6 mRNA Expression in DM Myocytes

**Figure 3:**
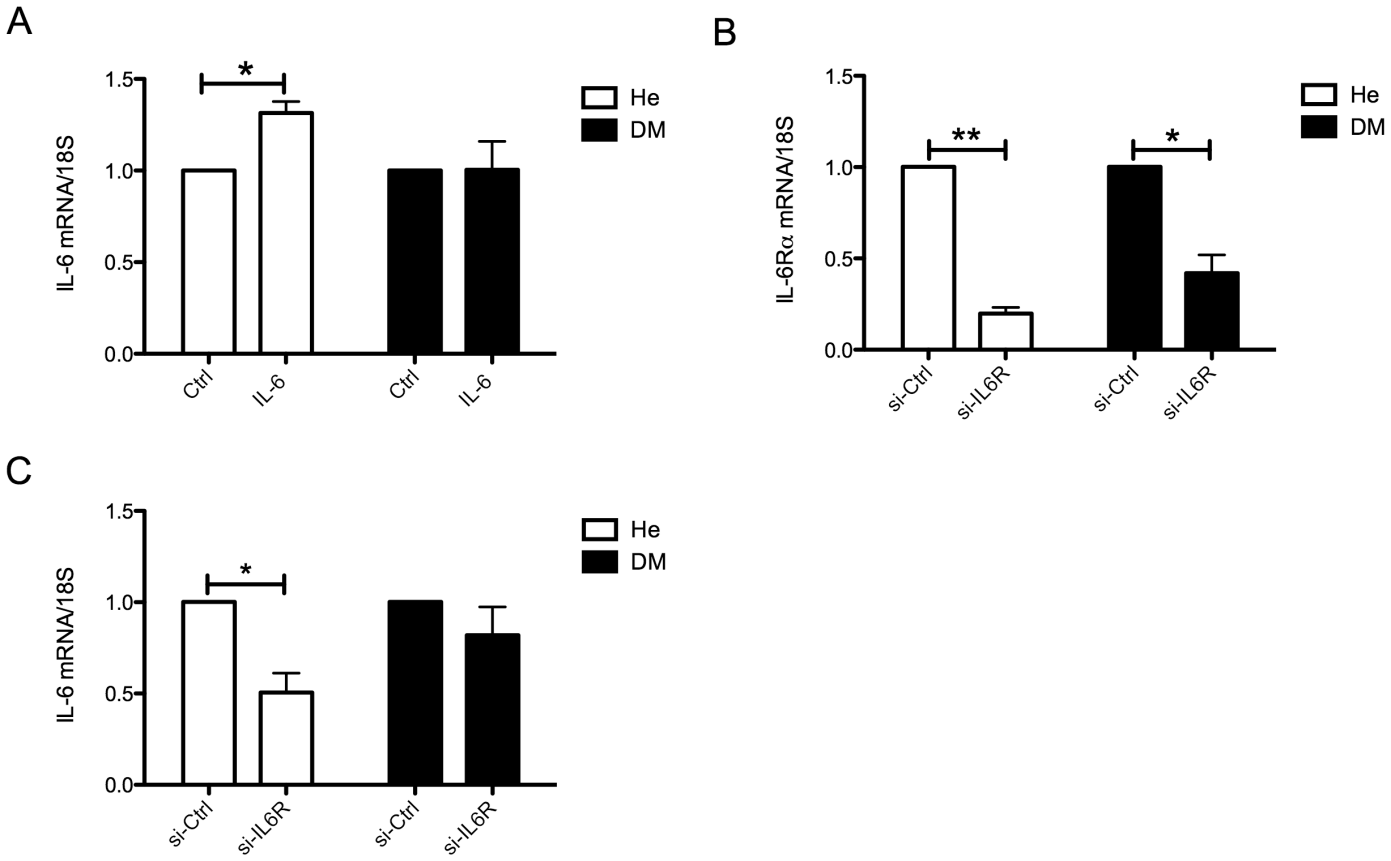
IL-6 induction of IL-6 mRNA in human myocytes. Muscle precursor cells derived from the vastus lateralis muscle of healthy (He), and obese people with type 2 diabetes (DM) (Table 3) (A) He myocytes (n = 5) and DM myocytes (n = 5) were treated with recombinant IL-6 or PBS for 60 minutes and IL-6 mRNA expression was assessed using qPCR. (B) Muscle precursor cells derived from healthy (n = 4), and obese people with type 2 diabetes (n = 4) were differentiated into myocytes and, during the last two days of differentiation transfected with siRNA targeting IL-6Rα. IL-6Rα knockdown was assessed using qPCR following 48 hrs of transfection. (C) IL-6 mRNA expression following IL-6Rα knockdown was assessed using qPCR following 48 hrs of transfection. Data were normalized to control treated samples within each cell subject and differences between groups were assessed using paired t-tests of dCT values. Data are mean ± SE *P<0.05, **P<0.01, ***P<0.001.

As mentioned in the introduction, IL-6 has been described to induce the expression of IL-6 in skeletal muscle cells [17], [18]. To investigate possible differences in IL-6 enhancement signaling between He and DM myocytes, we incubated human myocytes (Table 3) with recombinant IL-6 for 1 hour. This treatment induced IL-6 mRNA expression in He myocytes while DM myocytes did not respond (Figure 3A). We then targeted the endogenous IL-6 signaling by knocking down the expression of IL-6Rα, by transfecting the myocytes with siRNAs targeting IL-6Rα (Figure 3B). This would turn off the endogenous IL-6 stimulation of IL-6 and should therefore result in a reduced IL-6 expression. Indeed, 48 hours of knockdown of IL-6Rα resulted in a down-regulation of IL-6 in He myocytes but not in DM myocytes (Figure 3C). Notably, IL-6Rα was more reduced in the He myocytes (∼80%, P<0.01) than in the DM myocytes (∼60%, P<0.05), possibly partly explaining the lack of response in IL-6 expression. However, there was no difference between dCT-values between knockdown samples for the two groups (Figure S2) and IL-6 mRNA expression correlated with % knockdown of IL-6Rα in He myocytes (R^2^ = 0.92, P<0.05) but not in DM myocytes (R^2^ = 0.156, P<0.56) (Figure S2), indicating that the lack of changes in IL-6 expression following IL-6R knockdown in DM myocytes is likely to reflect a deficient regulation of IL-6 signaling. Due to a limitation of material, these experiments were performed on a different set of cells than the ones presented above and the obese group was not included. This data indicate that even though STAT3 phosphorylation in response to IL-6 is increased, endpoint gene expression activation fails in DM myocytes, possibly suggesting that the reduced SOCS3 activation and increased STAT3 activation is part of a compensatory mechanism for a reduced IL-6 signaling, i.e. an IL-6 resistance.

**Table 3:** Subject characteristics for primary muscle cell cultures, human study 2.

Subjects	Healthy	Type 2 diabetes
N	5	5
Gender	Male	Male
Age	65.2 (±5.4)	60.6 (±12.6)
BMI	24.3 (±3.8)	32.4 (±2.7)**
0 h Glucose (mmol/l)	5.1 (±0.6)	9.2 (±2.7)**
2 h Glucose (mmol/l)	5.3 (±1.7)	12.8 (±4.7)*

T-tests provided P values for people with type 2 diabetes were relative to healthy control subjects;

* P<0.05;

** P<0.01;

*** P<0.001. Values are mean ± SD.

### Resistance to IL-6 Induced AMPKα2 Activity in DM Myocytes

**Figure 4:**
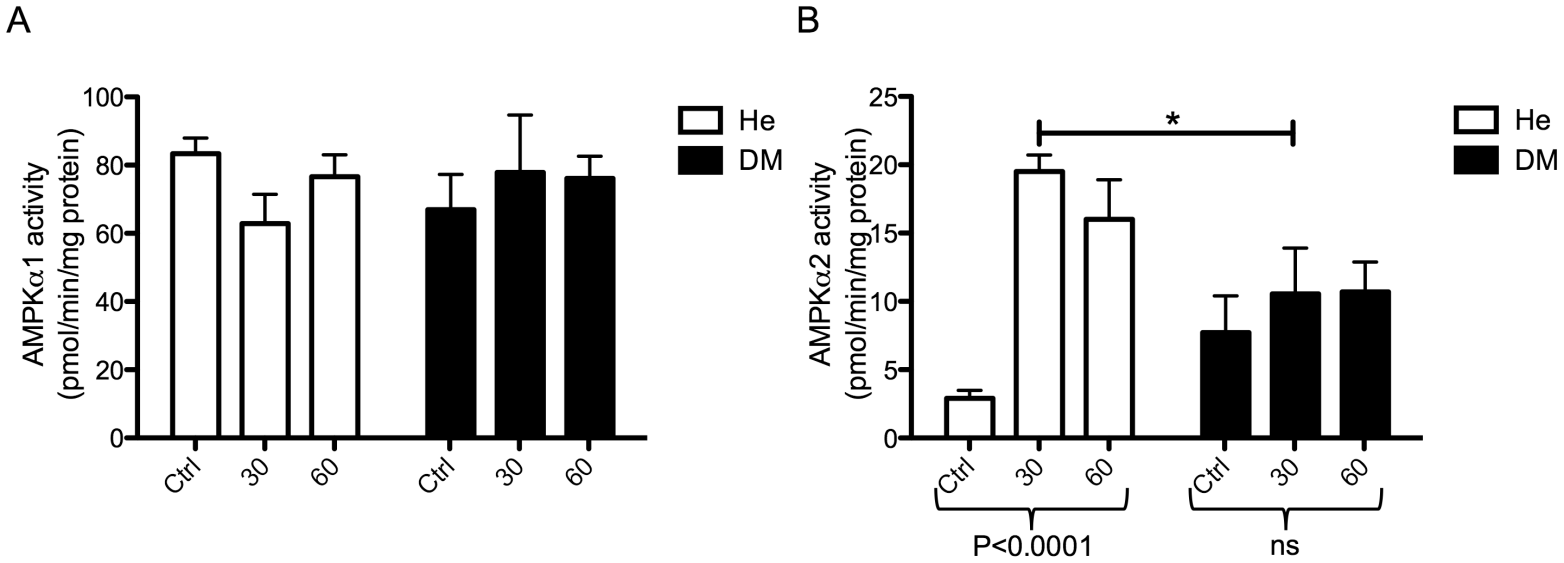
AMPK activity in response to IL-6 in myocytes from healthy and people with type 2 diabetes. Muscle precursor cells derived from the vastus lateralis muscle of healthy (He) (n = 5), and obese people with type 2 diabetes (DM) (n = 5) (Table 3), were differentiated into myocytes and were treated with recombinant IL-6 for 30 or 60 minutes. Controls (Ctrl) were treated with PBS for 60 minutes. Protein was isolated and AMPK activity was measured (A) AMPK α1 activity in response to IL-6 was compared between groups by a two-way ANOVA. (B) AMPK α2 activity in response to IL-6 was compared between groups by a two-way ANOVA. While there was no effect of group, there was an interaction (P<0.01) and thus Bonferroni post-tests were performed. Effect of treatment within groups was performed by one-way ANOVAs. Data are mean ± SE. *Results from Bonferroni post-tests, relative to He myocytes; *P<0.05, **P<0.01, ***P<0.001.

It has previously been shown that IL-6 specifically increase AMPKα2 activity [19], [20]. Therefore, to further evaluate the potential consequences of IL-6 resistance in type 2 diabetes, we assessed AMPK activity in response to 30 and 60 minutes of exposure to recombinant IL-6 in He myocytes and DM myocytes (Table 2). While no effect was observed on AMPKα1 activity in either of the cell groups (Figure 4A), we found a substantial increase in AMPKα2 activity in He myocytes (P<0.0001) but not in DM myocytes (Figure 4B). These data further emphasize an occurrence of an IL-6 resistance in DM myotubes, which may have consequences for the regulation of muscle metabolism.

### No Difference in Fusion Capacity between Myocytes from Healthy, Obese and People with Type 2 Diabetes

IL-6 has been shown to promote myogenesis [29] and SOCS3 promotes myogenic differentiation by down regulating the STAT3 signaling pathway [32]. Thus, we evaluated the myogenic status of muscle precursor cells isolated from healthy (He), obese (Ob) or people with type 2 diabetes (DM) (Table 1) differentiated into myotubes *in vitro*. Cells were stained for Myosin-2, widely utilized as a marker for mature myocytes and DAPI (Figure S3) and fusion index was determined by dividing the amount of DAPI positive cells within Myosin-2 positive myotubes with the total amount of DAPI positive cells. The same size of photograph was obtained from each slide and all DAPI positive cells in the photograph were counted. The average counted cells/photograph was 448±124 for He myocytes; 415±102 for Ob myocytes and 471±150 for DM myocytes (Figure S3). While phase contrast microscopy indicated that the majority of cells were fused into myotubes (data not shown), the average fusion index was determined to be 25%, suggesting that cell populations included both immature and mature myotubes. There was no significant difference in fusion index between the groups, possibly indicating that a “diabetic environment” or stimulating chronically with IL-6 might be necessary for the potential differences in differentiation capacity between the groups to transpire.

## Discussion

We have identified an abnormal IL-6 signaling in differentiated muscle precursor cells isolated from people with obesity and type 2 diabetes. We observed a reduced protein expression of IL-6Rα in skeletal muscle of people with obesity and with and without type 2 diabetes. This down-regulation of IL-6Rα protein was also found in Ob but not DM myocytes. However, further downstream, an abnormal STAT3/SOCS3 protein signaling response was established in DM but not Ob myocytes. Despite an increased pSTAT3 response, DM myocytes appeared resistant to IL-6 induced IL-6 gene expression and AMPKα2 activation. Confocal imaging and immunostaining for myosin 2 suggested equal differentiation status in all three cell groups.

The differences we observe in IL-6 response between He, Ob and DM myocytes, suggest that both obesity and type 2 diabetes results in modifications in satellite cell gene regulation, and that these alterations are stable enough to persist through multiple cell divisions and during differentiation into mature myocytes. Indeed, previous studies have reported donor specific responses to insulin stimulation [24]–[27] and we previously demonstrated that an inflammatory phenotype was conserved in isolated human myocytes [27]. As the regulation of IL-6 might be substrate dependent, it is possible that the difference in IL-6 response observed in the present study could reflect an acceleration of epigenetic modifications in obesity and type 2 diabetes. Nutritional environment has been shown to over time induce epigenetic modifications [33], causing stable alterations in genome structure.

Obesity is a major risk factor for developing type 2 diabetes and the majority of people with type 2 diabetes are obese. It is tempting to regard obese people as an intermediate group and obesity as a pre-state to diabetes. However, not all obese people develop diabetes, making it difficult to interpret a comparison with obese people that *have* developed diabetes. Nevertheless, we included muscle precursor cells from obese people in some of our experiments and observed a reduced protein expression of IL-6Rα, as well as a reduced protein expression of IL-6Rα in response to IL-6, while the STAT3/SOCS3 response seemed to be normal (although baseline SOCS3 levels tended to be increased). With our current data, it is not possible to conclude whether the dysregulation of IL-6 signaling in obesity and type 2 diabetes is linked or whether it represent two independent phenomena. Interestingly, IL-6Rα was down regulated in DM tissue but not in DM myocytes, while being down-regulated in Ob tissue *as well as* in Ob myocytes. This could indicate that the underlying mechanisms for down regulation of IL-6Rα *in vivo* differ between obesity and type 2 diabetes. We speculate that Ob myocytes have developed an increased negative control system of IL-6 signaling as a protection against the elevated levels of circulating IL-6 occurring in obesity [6]. We argue that this idea is supported by our finding that IL-6Rα is firmly down regulated at protein level in He myocytes as a response to prolonged (120 min) IL-6 administration. In contrast, DM myocytes rather seem to have lost control over IL-6 signaling, signified by an absent down-regulation of IL-6Rα, increased pSTAT3/STAT3 activation and a lack of up-regulation of SOCS3 in response to IL-6. Yet, end-point signaling such as IL-6 mRNA induction and AMPKα2 activation was deficient in another set of DM myocytes. Interestingly, we have previously demonstrated that pharmacological activation of AMPKα2 using the AMPK activator A769662 (100 µM) did not differ between any of the groups [27]. Whether this discrepancy in response is related to dose (we used 100 nM of IL-6), exposure time (A769662 was incubated with cells for 4 hours) or simply activation through separate pathways, remains elusive.

It is possible that the sensitized STAT3 phosphorylation accompanied by reduced SOCS3 activation in DM myocytes is a dysregulation compensating for other diabetes related alterations in cell signaling in skeletal muscle. For example, we recently demonstrated that several miRNAs, including two of the myomiRs (muscle specific miRNAs that regulate muscle cell differentiation) are differentially expressed in skeletal muscle of people with type 2 diabetes compared to healthy controls [34]. As SOCS3 suppression of STAT3 activity is an important mechanism during muscle differentiation [32], we assessed in the current study whether there was a difference in differentiation capacity of the different cell groups. Whereas we did not observe any difference in myosin-2 protein expression at the time point when myotubes were formed and we performed our experiments, it is possible that other markers, at other stages of differentiation are altered.

A dysfunctional SOCS3 regulation in myocytes of people with type 2 diabetes is of interest from a broader perspective, as members of the SOCS protein family not only regulate cytokine signaling but also has been shown to inhibit insulin receptor signal transduction [15], [35], [36]. The SOCS3 connection between IL-6 and insulin signaling could be an explanation to the observation that mice, when infused with high doses of IL-6 (0.5 µg/h), induced insulin resistance in skeletal muscle [37]. In support of this idea, another study demonstrated that lower doses of IL-6 (16 ng/h), corresponding to levels occurring in diabetic humans, limits the inhibiting effect on insulin signaling to liver, a tissue with a much higher expression of SOCS3 than muscle, while muscle remained unaffected [38]. We were not able to detect any significant differences in basal SOCS3 protein expression in the cell groups. However, Rieusset et al. previously demonstrated that SOCS3 mRNA levels were up regulated in skeletal muscle of people with type 2 diabetes but not in muscle of people with obesity only, despite comparable elevated plasma levels of IL-6 [39]. Taken together, regulation of the IL-6/STAT3/SOCS3 pathway in muscle cells seems to change with obesity, possibly due to alterations in circulating cytokine levels and composition. It remains elusive whether the additional changes to this pathway contributes to the manifestation of type 2 diabetes or represent secondary effects of the disease.

Acute stimulation with IL-6 has been shown to increase fatty acid turnover *in vivo* [40] and AMPKα2 activity [19], [20], fat oxidation [40]–[42] and glucose uptake [22], [40], [41], [43] *in vitro* in skeletal muscle cells. Thus, a deficient IL-6 signaling might have severe consequences for the regulation of metabolic rate in skeletal muscle. In the present study we demonstrated that IL-6 potently induces AMPKα2 activity in He myocytes, but not in DM myocytes derived from individuals with diabetes. Several studies have demonstrated that IL-6 stimulates fatty acid metabolism in rodent skeletal muscle by the activation of AMPK [19]–[21]. Thus, an IL-6 resistant muscle may suffer from a compromised energy regulation, independently of the insulin pathway. A similar deficiency has been reported for leptin signaling in myocytes derived from obese subjects in comparison to lean [44]. Here, myocytes from obese subjects failed to activate AMPK in response to leptin administration, accompanied by a corresponding deficiency in palmitate oxidation [44]. However, while IL-6 activated AMPKα2 8-fold in He myocytes in our study but had no effect on AMPKα1, leptin increased both AMPKα1 and AMPKα2 (approximately 2-fold) [44], suggesting that IL-6 is a more potent and specific stimulant of AMPKα2. It is therefore tempting to suggest that a deficiency in IL-6 signaling might have even more severe consequences for AMPKα2 downstream signaling than a deficiency in leptin signaling, possibly limiting both fatty acid oxidation and glucose uptake in the diabetic muscle. Indeed, IL-6 knockout mice develop moderate obesity, glucose intolerance and dyslipidemia at 9 months of age [45].

The role of IL-6 in muscle metabolism is further elucidated by the recent report that IL-6 stimulate insulin secretion via GLP-1 induction [11], suggesting that the increased levels of IL-6 associated with obesity and insulin resistance might be a compensatory mechanism (i.e. an increased IL-6 production is initiated to induce more insulin). However, this chronic exposure of elevated IL-6 levels might have severe effects on IL-6 signaling in skeletal muscle. A chronic IL-6 stimulation may interfere with the regulation of IL-6 signaling and indeed we here report a down regulation of the IL-6Rα receptor in skeletal muscle biopsies of people with obesity and a deficient downstream IL-6 signaling in DM myocytes.

Our data indicate that negative control of IL-6 signaling is increased in myocytes in obesity, whereas a dysfunctional IL-6 signaling is established further downstream of IL-6Rα in DM myocytes, resulting in a failure in activating gene expression and metabolic rate. Our data raises the idea that additional growth signaling pathways might be altered in skeletal muscle of people with type 2 diabetes. Further studies of these pathways in isolated myocytes might provide a substantial contribution to the current view of the muscular pathophysiology of type 2 diabetes mellitus.

## Materials and Methods

### Ethics Statement

Permission to undertake the human studies, from which muscle tissue and satellite cells were obtained, was approved by the Ethics Committee of Copenhagen and Frederiksberg Communities, Denmark (KF 01-141/04) (human study 1) and (H-C-2008-101) (human study 2). The participants were given oral and written information about the experimental procedures. All volunteers gave their written consent before participation. Both studies were carried out in accordance with the principles of the Declaration of Helsinki as revised in 2000.

### Human Studies

We utilized material from two previously described studies. Human study 1, was described previously [27] and was approved by the regional research ethics committee (H-C-2008-101). From this study, satellite cells were isolated from non-obese persons, and from obese persons with and without type 2 diabetes (n = 7 in each group, Table 2). From human study 2 (H-KF-01-141/04), additional satellite cell cultures were obtained. Subjects and study protocol have been described previously [6], [46]–[48]. One sub-set (non-obese persons or individuals that had type 2 diabetes n = 5 in each group, Table 3) was utilized for satellite cell isolations. Another subset of the samples (non-obese, obese persons or individuals that had type 2 diabetes n = 9–10 in each group, Table I) was utilized for protein analysis. The participants in both studies were divided into distinct groups according to BMI (≤30 or >30 kg/m^2^), categorized as non-obese or obese and, according to the WHO definition, normal glucose tolerant, impaired glucose tolerant and the diagnosis of type 2 diabetes.

### Satellite Cell Culture

Satellite cells were isolated from muscle biopsies as previously described [33]. Unless otherwise stated, all cell culture reagents were from Invitrogen, Carlsbad, CA, USA. Briefly, a muscle biopsy was obtained from vastus lateralis and minced and digested in 5 ml HamF10 media containing 50% trypsin EDTA (1×) and 5 mg collagenase IV (Sigma-Aldrich, St.Louis, MO, USA) and BSA (Sigma-Aldrich) in 37°C for 5 minutes. The digestion solution was inactivated in serum on ice and the procedure was repeated. The digestion solution was filtered and centrifuged at 800 g for 7 minutes. Following washing with HamF10, cells were pre-plated in a 60 mm culture plate, to diminish fibroblast contamination. After 3 hours incubation, the cell suspension was transferred to a 25 cm^3^ flask coated with matrigel (BD, Franklin Lakes, NJ, USA). The media, HamF10, 20% FBS, 1% Penicillin/Streptomycin and 1% Fungizone, was changed on day 4 after isolation and thereafter every second day. Cell cultures were expanded from the 25 cm^3^ flask to a 10 cm plate, which were assigned passage (P) 1. Thereafter, cells were split 1∶3 at 75% confluence. Experiments were performed on cells at P3–P4. For differentiation, cells were seeded in DMEM low glucose, 10% FBS, 1% Penicillin/Streptomycin and, upon confluence, media was changed to DMEM high glucose, 2% horse serum, 1% Penicillin/Streptomycin. At day 5 in low serum, the majority of the cells had fused into myotubes with polynucleated status. Cells were treated with human recombinant IL-6 (R&D systems, Minneapolis, MN, USA) for 30, 60 or 120 minutes at a concentration of 100 ng/ml, if not indicated otherwise in the figure legends and harvested in cell lysis buffer for AMPK activity or Western Blot analysis or Trizol for RNA and subsequent gene expression analysis.

### siRNA Transfection of Myocytes

Transfections on human myoblasts and myotubes were performed using siRNA pools consisting of four siRNA oligos specifically targeting four different sites of target mRNA (IL6Rα) (On Target Plus) (Dharmacon). The purpose of the siRNA pools is to use less of each oligo to minimize any off-target effects. Transfections were performed using lipofectamine 2000 (Invitrogen, Taastrup, Denmark), according to the manufacturer’s protocol, with 100 nM of siRNA, at day 3 of differentiation in antibiotic-free cell culture media (see above), for 48 h. A scrambled non-specific oligonucleotide (siRNA Scr) was used as control.

### Immunofluorescence

Myoblasts were differentiated in chamber slides pre-coated with poly-L-lysin and matrigel. At day 5 of differentiation, myocytes were permeablized and fixed with methanol at −20°C for 6 minutes. Non-specific binding sites were blocked in PBS containing 2% BSA over night at +8°C. Primary antibody targeting MYH2 (MF20, DSHB) was diluted 1∶50 in PBS containing 5% goat serum while secondary antibody (Alexa-fluor 488, Molecular Probes/Invitrogen) was diluted 1∶1000 in PBS. Primary and secondary antibody incubations were performed at 37°C for 60 and 30 min, respectively. Nuclei were stained with DAPI for 10 minutes at room temperature. Between all steps, cells were washed for 3×5 min with PBS at RT. Stained myocytes were mounted using Vectashield Mounting Medium and sealed with nailpolish. Myocytes were examined and photographed under a Zeiss LSM 710 on Axio Imager at 40× magnification. The same size of photograph was obtained from each slide and all DAPI positive cells in the photograph were counted. To estimate a fusion index, the number of DAPI positive cells within MYH2 positive myotubes was divided with the total number of DAPI positive cells.

### Western Blotting

Human muscle samples were homogenized using a Tissuelyser (Qiagen, Valencia CA, USA) in 50 mM Tris-HCl, pH 7.4, 150 mM NaCl, 1 mM EGTA, 1 mM EDTA, 0.25% NaDeoxycholate, 1% Triton X-100. Cell samples were harvested in lysis buffer (Cell signaling technology, Danvers, MA, USA)). Phosphatase inhibitor cocktail 1 and 2 (SigmaAldrich) and protease inhibitor complete mini (Roche) was added fresh to the buffer. Protein concentration was measured using a colorimetric protein assay (Bio-Rad) and samples were diluted in 5 × Laemmli buffer and boiled for 2 min before loading of 25 µg of muscle tissue lysates or 10 µg of cell lysates onto a 4%–12% gradient bis-Tris NuPage gel (Invitrogen). Protein was transferred onto a PVDF membrane using a semi-dry blotting system for 1 h and 30 minutes at 20 V (Invitrogen). The membrane was blocked for 1 h in 5% milk and incubated with primary antibody overnight at 4°C. Antibodies were against: IL-6Rα (Santa Cruz), STAT3, p-STAT3(tyr^705^), SOCS3 (all from Cell Signaling Technology). Blots were washed and incubated with secondary IgG HRP conjugated antibody (Cell Signaling Technology). Signal was detected using Supersignal West Femto Luminal/Enhancer Solution (Thermo Scientific) and subsequent exposure in a charge-coupled device camera (Bio-Rad). Blots were incubated in 0.5% Reactive Brown (Sigma Aldrich) for 15 min. Blots were analyzed and quantified using ImageQuant software, using reactive brown as a measure of total protein and for normalization for loading and transfer.

### AMPK Activity Assay

Protein lysates were performed and quantified as described above. AMPK activity was assessed as previously described [34]. Briefly, 100 µg of protein was immunoprecipitated with antibody specific to the α2 or α1 catalytic subunit of AMPK (Bethyl antibodies) and protein A/G Sepharose beads over night at 4°. Beads were washed five times, and the activity of the immobilized enzyme was assayed based on the phosphorylation of “SAMS” peptide (0.2 mmol/l) by 0.2 mmol/l ATP (containing 2 µCi [γ-32P] ATP) in the presence and absence of 0.2 mmol/l AMP. Label incorporation into the SAMS peptide was measured on a Beckman LS6500 scintillation counter.

### RNA Isolation and Quantitative Real-time PCR

Skeletal muscle biopsy samples were homogenized in TRIzol (Invitrogen) using a Tissuelyser (Qiagen) and total RNA was isolated according to the manufacturer’s protocol. Total RNA was dissolved in nuclease-free water and quantified using a Nanodrop ND 1000 (Saveen biotech ApS, Århus, Denmark). Total RNA (0.5 µg) was reverse-transcribed using cDNA high capacity kit (Applied Biosystems) according to the manufacturer’s protocol. CDNA samples were loaded in triplicates and quantitative real-time PCR (qPCR) was performed using an ABI-PRISM 7900 Sequence Detection system (Applied Biosystems, Foster City, CA, USA) according to the manufacturer’s protocol. IL-6 Primers and MGB probe were designed using Primer Express software (Applied Biosystems) (IL-6 forward primer: 5′-ctgcagaaaaaggcaaagaatctag, IL-6 reverse primer: 5′-tctgtgcctgcagcttcgt, IL-6 MGB probe: 5′-cacccctgacccaac). IL-6Rα primers were designed using Roche Universal probe library (Roche), IL-6Rα forward primer: 5′agtaccactgcccacattcc, IL-6Rα reverse primer: 5′-cagcttccacgtcttcttga. A pre-optimized assay for 18 S rRNA was used as endogenous control (Applied Biosystems). To assess amplification efficiency, two fold dilutions series were performed for IL-6, IL-6Rα and 18 S. Based on these, cDNA was diluted 1∶10 for IL-6 and IL-6Rα and 1∶200 for 18 S, to ensure linear amplification. RefSeq Accession numbers are: IL-6: NM_000600.3, IL-6Rα: NM_000565.2.

### Statistical Analysis

Statistical analysis was performed using GraphPad Prism software. All data are presented as mean ± SE. Two-way Anova (Repeated measures) was used for comparisons of time and metabolic group. Comparisons of pairs were performed (He vs Ob, He vs DM and Ob vs DM). When relevant and as specified in figure legends, Bonferroni post-hoc tests were performed. QPCR data were presented normalized to control treated samples within each cell subject and differences between groups were assessed using t-tests of dCT values. When relevant and as specified in figure legends and/or results, one-way Anova was utilized to assess regulation over time *within* each cell group. Sample size and significance level for each analysis are as stated in figure legends. A P-value of 0.05 was considered significant.

## Supporting Information

Figure S1 **IL-6R**α **protein expression in human muscle tissue.** All blots for the data presented in Figure 1A are shown. Samples are from non-obese, normal glucose tolerant subjects (NGT), obese normal glucose tolerant subjects (Ob), non-obese subjects with type 2 diabetes (DM) and obese subjects with type 2 diabetes (DMO).
(TIFF)

Figure S2 **IL-6R**α **knockdown in He vs DM myocytes**. (A) Comparison between dCT values in samples treated with IL-6Rα for the two groups. (B) Comparison between IL-6Rα knockdown and IL-6 mRNA expression. Linear regression analyses were performed on the data presented in Figure 3B and 3C.
(TIFF)

Figure S3 **Differentiation status of myocytes derived from healthy, obese or people with type 2 diabetes.** Muscle precursor cells derived from the vastus lateralis muscle of healthy, obese and obese people with type 2 diabetes (Table 2) were differentiated into myocytes on chamber slides. (A) Myocytes were immunostained for Myosin-2 and counterstained with DAPI. (B) The amount of DAPI positive cells within Myosin-2 positive myotubes was divided with the total number of DAPI positive cells, to estimate a fusion index. Data are mean ± SE (n = 5–6 in each group).
(TIFF)
