# Supplementary figures and images for: Satellite Cells Derived from Obese Humans with Type 2 Diabetes and Differentiated into Myocytes In Vitro Exhibit Abnormal Response to IL-6
Source: PLoS One. 2012 Jun 26;7(6):e39657. doi: 10.1371/journal.pone.0039657 (PMC3383673; doi:10.1371/journal.pone.0039657)

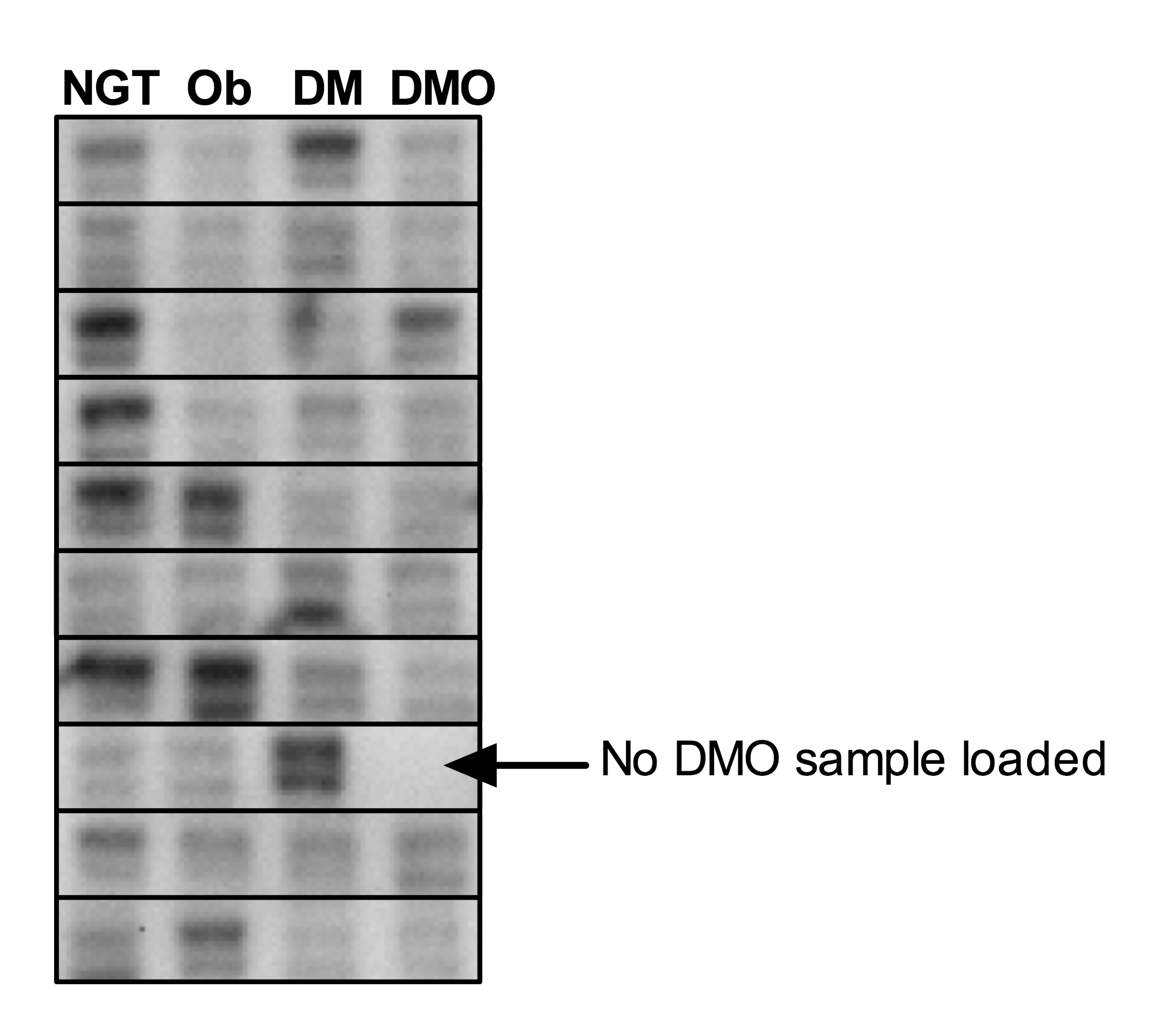

Supplement: Figure S1 — IL-6Rα protein expression in human muscle tissue. All blots for the data presented in Figure 1A are shown. Samples are from non-obese, normal glucose tolerant subjects (NGT), obese normal glucose tolerant subjects (Ob), non-obese subjects with type 2 diabetes (DM) and obese subjects with type 2 diabetes (DMO). (TIFF) [file pone.0039657.s001.tiff]

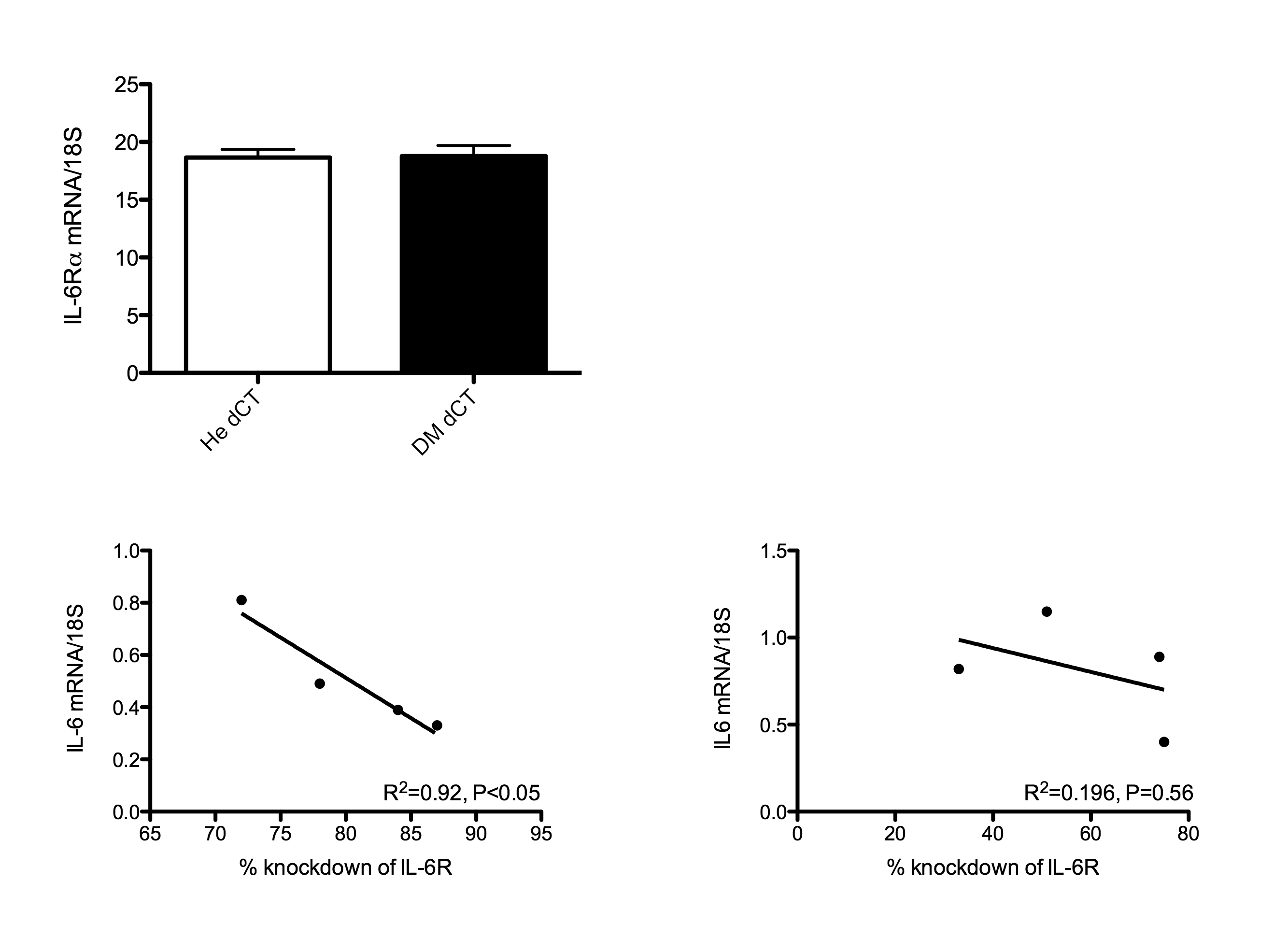

Supplement: Figure S2 — IL-6Rα knockdown in He vs DM myocytes. (A) Comparison between dCT values in samples treated with IL-6Rα for the two groups. (B) Comparison between IL-6Rα knockdown and IL-6 mRNA expression. Linear regression analyses were performed on the data presented in Figure 3B and 3C. (TIFF) [file pone.0039657.s002.tiff]

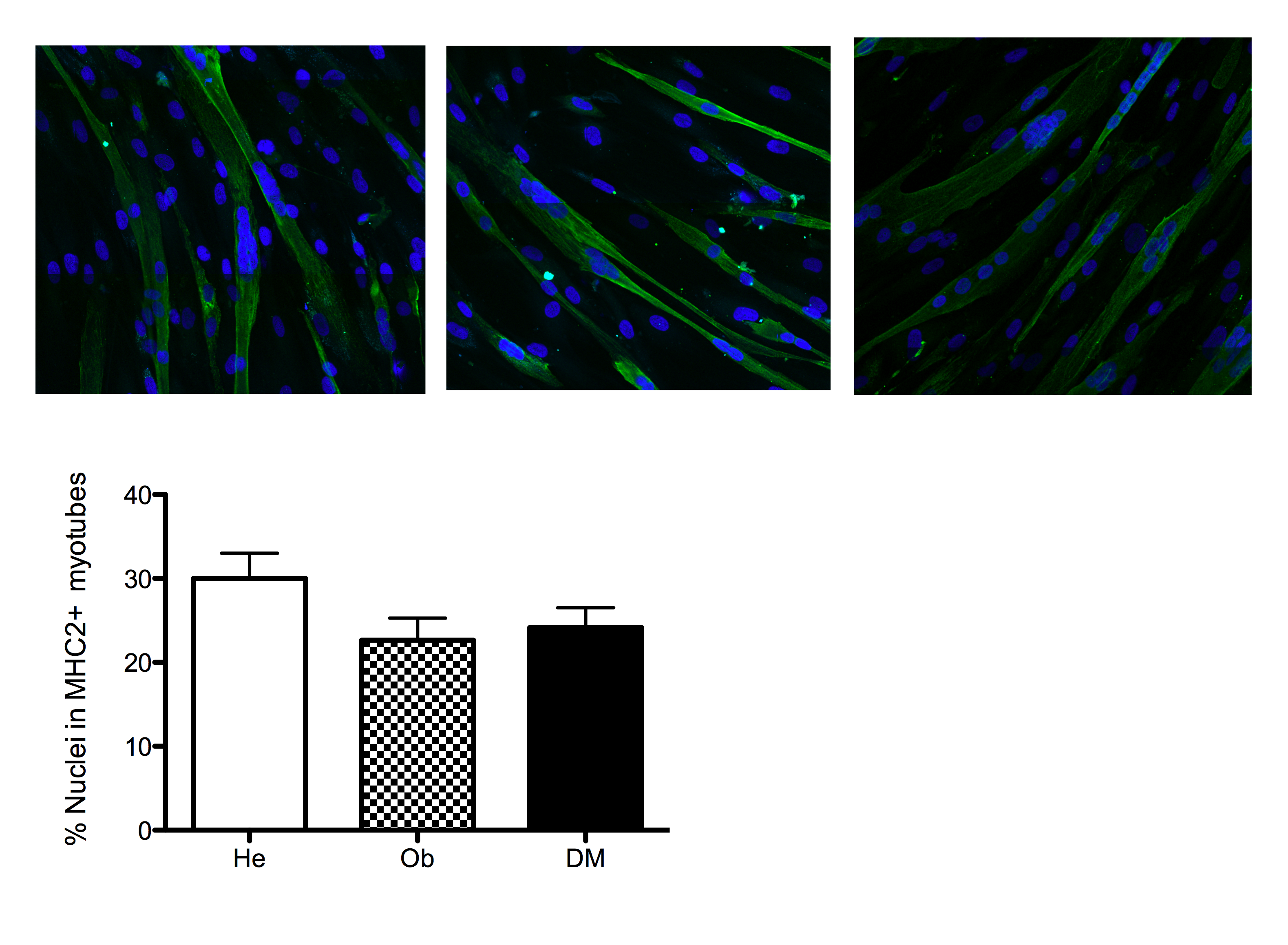

Supplement: Figure S3 — Differentiation status of myocytes derived from healthy, obese or people with type 2 diabetes. Muscle precursor cells derived from the vastus lateralis muscle of healthy, obese and obese people with type 2 diabetes (Table 2) were differentiated into myocytes on chamber slides. (A) Myocytes were immunostained for Myosin-2 and counterstained with DAPI. (B) The amount of DAPI positive cells within Myosin-2 positive myotubes was divided with the total number of DAPI positive cells, to estimate a fusion index. Data are mean ± SE (n = 5–6 in each group). (TIFF) [file pone.0039657.s003.tiff]
